# Assessment of changes in optical and mechanical properties and adverse effects of over-the-counter bleaching agents: An *in vitro* study

**DOI:** 10.4317/jced.62498

**Published:** 2025-02-01

**Authors:** Murilo Navarro de Oliveira, Helena Benatt do Nascimento Alves, Fabiana Evangelista Lerner, Murilo Guimarães Campolina, Caio Luiz Lins-Candeiro, Walbert de Andrade Vieira, Marcelo Bighetti Toniollo, Gisele Rodrigues da Silva, Luiz Renato Paranhos

**Affiliations:** 1 PhD student, Postgraduate program in dentistry, Faculty of Dentistry, Universidade Federal de Uberlândia (UFU), Brazil; 2 Undergraduate student, Faculty of Dentistry, Universidade Federal de Uberlândia, Brazil; 3 Centro Universitário das Faculdades Associadas de Ensino (UNIFAE), Brazil; 4 Dental School of Rio Verde, Universidade de Rio Verde (UniRV), Brazil; 5 Department of Operative Dentistry and Dental Materials, Faculty of Dentistry, Universidade Federal de Uberlândia (UFU), Brazil; 6 Department of Preventive and Social Dentistry, Faculty of Dentistry, Universidade Federal de Uberlândia (UFU), Brazil

## Abstract

**Background:**

This *in vitro* study evaluated the impact of over-the-counter (OTC) products on the optical properties (color and gloss changes) and mechanical properties (shear bond strength) of tooth enamel subjected to a simulated orthodontic treatment.

**Material and Methods:**

80 bovine teeth were selected and subjected to a staining protocol and initial color assessments. Then, orthodontic brackets were bonded on the center of the clinical crown of each sample, and teeth underwent the bleaching protocols. The samples were allocated to five groups (n=16): Conventional in-office bleaching 35% Hydrogen peroxide - positive control (HP35); Dentifrice with 2% HP (DHP); Mouthwash with 2.5% HP (MouHP); Paint-on with 6% HP (PON); Conventional dentifrice without HP - negative control (DWHP). After receiving the bleaching protocol, the samples underwent the shear bond strength test for orthodontic bracket removal. The remaining resin was then removed from the sample surfaces using multi-blade burs. Data was analyzed with Kruskal-Wallis test or one-way ANOVA, with 5% significance.

**Results:**

Regarding color change, multiple comparison analyses showed significant differences (*p*< 0.05). The HP35 and MouHP groups showed a significantly greater color change compared to the DWHP and DHP groups for both ΔE00 and ΔEab values, while the PON group did not differ significantly from the other groups. The statistical test did not detect a significant difference in post-bleaching gloss increase between the experimental groups (*p*=0.20). In addition, it did not detect a significant difference between the experimental groups for shear assessment (*p*=0.14).

**Conclusions:**

Except for dentifrices, the color change outcomes of the evaluated OTC bleaching treatments were as satisfactory as in-office bleaching, without differences between groups for gloss change. The study suggests that the evaluated OTC products did not influence the bond strength of orthodontic brackets to teeth.

** Key words:**Hydrogen Peroxide, In Vitro Technique, Tooth Bleaching, Tooth Bleaching Agents.

## Introduction

Dental bleaching and orthodontic alignment rank among the primary aesthetic concerns of patients seeking dental care (1,2). Beyond improving appearance, changes in tooth color and position have been shown to positively influence social perception, attractiveness, intellectual ability, and relationship satisfaction (3). As a result, the combination of orthodontic treatment and dental bleaching is frequently requested by patients aiming to achieve both ideal tooth alignment and whiteness simultaneously (4).

With the rise in availability of over-the-counter (OTC) whitening products, an increasing number of patients are turning to these more convenient and affordable options during orthodontic treatment. Once dental crowding is corrected, dissatisfaction with tooth color often intensifies (1), prompting the use of OTC bleaching agents to improve dental aesthetics without requiring professional supervision. These products, which do not require a prescription, generally contain lower concentrations of hydrogen peroxide compared to in-office treatments (5). They are marketed in various forms, including gels, toothpastes, mouthwashes, Whitestrips, and paint-on formulations. Although some studies have demonstrated that OTC products can effectively whiten teeth, the overall body of evidence remains limited. Additionally, the safety and efficacy of these products vary widely depending on their active ingredients and formulation, with certain agents posing a risk of enamel damage (6).

Whitening gels, particularly those containing hydrogen peroxide, have the ability to penetrate both enamel and dentin, allowing the bleaching effect to extend beyond the immediate application site. As a result, the whitening process can affect areas beneath and around orthodontic brackets, not just the exposed tooth surface, providing a successful treatment during orthodontic treatment, yielding favorable aesthetic outcomes (7).

However, the effects of OTC bleaching products on orthodontic brackets and their adhesion to enamel remain uncertain. The variability in product formulations, peroxide concentrations, and active ingredients, particularly in products obtained without professional supervision, raises concerns about their safety and effectiveness (5,6). Another critical issue is the potential impact of bleaching agents on the adhesive bond strength of orthodontic brackets (8). While the literature offers conflicting evidence on the clinical relevance of reduced bond strength at the bracket-enamel interface following bleaching (8,9), there is a notable lack of studies specifically addressing the effects of OTC bleaching products on this interaction.

This research gap highlights the need for further investigation into the influence of OTC bleaching agents on orthodontic outcomes. Therefore, the present study aimed to evaluate how bleaching products containing OTC hydrogen peroxide act on the optical and mechanical properties of enamel subjected to orthodontic treatment. The null hypotheses tested were: 1) OTC products do not affect enamel color or gloss, and 2) OTC products do not alter the shear bond strength of orthodontic brackets bonded to enamel.

## Material and Methods

-Study design and sample size

The study design followed the CRIS (Checklist for Reporting In vitro Studies) tool guidelines (10), according to the recommendations for *in vitro* studies. The eighty bovine tooth samples originated from slaughterhouse donations, exempting them from the ethical committee approval for animal testing. All tests followed the scientific requirements and research protocols established by the World Medical Association Declaration of Helsinki. The sample size calculation was performed using the G*Power software (latest ver. 3.1.9.7; Heinrich-Heine-Universität Düsseldorf, Düsseldorf, Germany; http://www.gpower.hhu.de/. The calculation was based on the experimental design, using the following parameters: 5% two-tailed significance level (α = 0.05), 95% confidence interval, 90% statistical power (β = 0.10), 1:1 sample allocation ratio to experimental groups, and a large estimated effect size (d = 0.80), indicating the need for at least 16 samples in each group, totaling 80 samples (11).

-Sample selection and preparation

After extraction, the bovine incisors were cleaned with periodontal curettes and stored in distilled water. Teeth selection followed the eligibility criteria: 1. Healthy permanent incisors; 2. Teeth without fractures in the coronal region; 3. The absence of macroscopically visible enamel cracks. The excluded teeth were discarded properly as biological materials. The initial color assessment involved cleaning the samples, polishing them with pumice, and extrinsically staining them by immersion them in black coffee immersion for three days. The solution was replaced every 24 hours and prepared according to the manufacturer’s instructions by diluting two teaspoons of powder (Nescafé®, Nestlé, São Paulo, SP, Brazil) in 100 ml of boiling water (11). After completing staining protocol, the samples were cleaned in running water to remove the excess coffee and dried at room temperature for six hours. A diamond disc (American Burrs, Palhoça, SC, Brazil) was used to remove the roots, and the samples were stabilized by the palatal surface in a colorless acrylic resin base (VIPI FLASH, Pirassununga, SP, Brazil) made with a polyvinyl chloride (PVC) tube (Conexões Tigre, Joinville, SC, Brazil) of 12 mm in diameter, completely exposing the buccal aspect of the crown.

-Color evaluation

A reflectance spectrophotometer (Ci64UV, X-Rite, Grand Rapids, MI, USA) evaluated sample colors. This device has an opening diameter of 4 mm, and the readings occurred at a 2° observation angle and illuminant D65. The LAB system coordinates of the Commission Internationale de L’Eclairage (CIE) were recorded. This system consists of luminosity (L* coordinate) and a* (red-green axis) and b* (yellow-blue axis) chromaticity coordinates. The color was measured at baseline (T0) and 72 hours after finishing the bleaching treatment (T1); the samples were stored in distilled water during that period. Color measurements occurred in triplicate on a white background (ColorChecker grayscale, X-Rite, Grand Rapids, MI, USA, L*white = 95.2, a*white = 21.2, b*white = 50.3), and the data analysis used the mean values of the three assessments. The color change result (difference between baseline-T1) was calculated with two methods (12,13): CIELab:ΔEab = [(ΔL*)2 + (Δa*)2 + (Δb*)2].1/2; CIEDE2000 formula: ΔE00 = [(ΔL/kLSL)2 + (ΔC/kCSC)2 + (ΔH/kHSH)2 + RT (ΔC × ΔH/SC × SH)]. As for color change assessment, the perceptibility thresholds were established at ΔEab=1.2 and ΔE00=0.8, while acceptability thresholds were set at ΔEab=2.7 and ΔE00=1.77 (12).

-Gloss assessment 

The gloss of the samples was measured using a glossmeter (3NH Global, NHG60M, Shenzhen, China) in GU. An angle of 60o was applied to evaluate the gloss at the center of the sample and a 2 mm x 2 mm square area. The mean of the three measurements per sample was calculated on T0 and T1 (72 hours after the treatment).

-Bracket bonding

Eighty metal brackets with an area of 6 mm2 (Kirium U1R Roth 022; Abzil 3M, São José do Rio Preto, SP, Brazil) were cemented on the center of the samples. The region delimited by the mold was etched with 37% phosphoric acid (Alpha-Etch; Nova DFL, Rio de Janeiro, RJ, Brazil) for 30 seconds according to the manufacturer’s specifications, then washed with water for 30 seconds and dried by air spray. Next, an adhesive layer (Scotchbond; 3M Unitek, St. Paul, MN, USA) was light-cured for 20 seconds according to the manufacturer’s instructions (Valo; Ultradent Products, Inc., South Jordan, UT, USA) and applied. A composite resin layer (Transbond™ XT; 3M Company, St. Paul, MN, USA) was then spread under the metal bracket base placed on the tooth surface aided by orthodontic tweezers. After a quick three-second light-curing, the excess resin was removed from the dental surface aided by a spatula (Golgran, São Caetano do Sul, SP, Brazil). Then, a final light-curing was performed for 20 seconds.

-Bleaching treatment

A blind evaluator allocated the samples to five groups using randomization software (www.random.org). The samples were stored in distilled water until to subjected to bleaching agents. The description of the bleaching protocols is shown in  Table 1.

-Bracket removal and adhesive strength assessment

All samples were subjected to a mechanical shear test in a machine (EMIC DL1000, São José dos Pinhais, PR, Brazil). This equipment was used with a 1kN cell and an active chiseled tip placed between enamel and resin, and the speed was 0.5 mm/min. The maximum force for rupture was recorded in Newtons (N). After bracket removal, a multi-blade bur helped eliminate the excess surface resin to remove the orthodontic adhesive.

-Data analysis

A blinded participant performed the statistical analysis. The data were analyzed with R software, version 4.3.0, aided by the stats, rstatix, and ggplot2 packages. The Shapiro-Wilk and Bartlett tests evaluated the normality and homogeneity of data variance, respectively. The data from shear analyses and color change presented a non-normal distribution (*p* < 0.05); hence, Kruskal-Wallis with Dunn post-hoc test and Bonferroni correction was applied. ANOVA one-way and Welch with Games-Howell post hoc test analyzed the post-bleaching gloss changes. All analyses used a 5% significance level.

## Results

-Color and gloss assessment

The analyses included the data from all specimens. The Kruskal-Wallis test detected a significant difference between the groups (ΔE00 – *p* = 0.000069). Multiple comparison analyses showed significant differences (*p*< 0.05) between groups DHP vs. MouHP, DWHP vs. MouHP, DHP vs. HP35, and DWHP vs. HP35 ( Table 2). ANOVA did not detect a significant difference in the post-bleaching gloss increase between the experimental groups (F(4.75) = 1.53, *p* = 0.20) ( Table 3).

-Shear test

The shear test excluded six specimens from the analysis due to technical failure. The Kruskal-Wallis test did not detect significant differences between the experimental groups (*p* = 0.14) ( Table 4).

## Discussion

When comparing OTC whitening products, such as toothpastes containing low concentrations of hydrogen peroxide, their whitening effects are mainly achieved through the mechanical removal of extrinsic stains rather than chemical bleaching. This mechanical action, driven by abrasive ingredients, explains why the color change observed in the toothpaste groups was comparable to previous studies (17,18). Additionally, research suggests that whitening toothpastes do not necessarily cause more abrasiveness to the enamel than regular toothpastes (18).

The findings of this study align with other *in vitro* studies that investigated OTC whitening products without the presence of orthodontic brackets (20,21). These studies similarly observed significant tooth color changes following the use of over-the-counter agents. Importantly, the presence of orthodontic brackets in this study did not hinder the overall effectiveness of the whitening treatments, supporting the notion that bleaching agents can still achieve noticeable results even when brackets are present (9).

However, there may be localized differences in color beneath and around the brackets. While some studies suggest these differences can be statistically significant, they are often not clinically perceptible (23,24). Despite these minor differences, all experimental groups in this study demonstrated a mean ΔE00 value higher than the threshold for visible color change (ΔE00>1.77), indicating that the whitening effects were visible across all groups (12).

The whitening effects observed in this study can also be partially attributed to the abrasives found in the dentifrices, such as calcium pyrophosphate or hydrated silica, which are effective in removing extrinsic stains (25). Although this study did not assess long-term color stability, future research should focus on evaluating how well OTC products like MouHP and PON maintain their whitening effects over time, as both performed similarly to the 35% hydrogen peroxide treatment (HP35) in the short term.

In particular, the mouthwash group (MouHP) showed color change results comparable to the 35% hydrogen peroxide (HP35) group. This mouthwash contains 2.5% hydrogen peroxide, a concentration slightly higher than that in the toothpaste group (DHP), and also includes phosphoric acid, which likely contributed to its superior whitening effect. Although the exact concentration of phosphoric acid is not provided, its inclusion may have facilitated the removal of surface stains by breaking down surface deposits (26). This could explain why the mouthwash demonstrated a more pronounced color change than the toothpastes.

Gloss change is related to the light reflected on the enamel surface, and increasing this value benefits esthetic perception because it may give the impression of a bleached tooth (27). All groups showed a positive color change in sample gloss without statistical differences between the groups, favoring the color change findings.

The shear assessment did not show statistical differences between the groups suggesting that the evaluated bleaching agents did not harm the bond strength of brackets. These findings agree with other *in vitro* studies (28); however, the present investigation treated the teeth for a controlled time, which would not necessarily occur if patients purchased these products indiscriminately and used them without professional supervision.

The decreased bond strength of brackets with orthodontic cement may cause their detachment, promoting patient discomfort and the need for additional repair and bonding sessions (29). However, the means obtained in all groups reached the minimum adhesion results recommended in the literature: 50-70 N (29). The findings suggest that bleaching agent action cannot harm adhesion to the point of clinically impairing orthodontic treatment development. We also suggest further studies to evaluate whether the prolonged use of these agents may cause adverse effects on material shear bond strength.

A limitation of laboratory studies with OTC products is the impossibility of evaluating gingival irritation caused by these agents. Considering the absence of a dentist’s follow-up, the bleaching protocol is not customized, promoting the risk of inadequate product application. The direct contact between the bleaching agent and soft tissues may cause inflammatory reactions because hydrogen peroxide is toxic to the fibroblasts of the gingival tissue, leading to an inflammatory reaction (30).

The agents effectively changed the color of samples, indicating the possibility of further clinical studies, even in patients undergoing orthodontic treatment who wish to perform tooth bleaching, to correctly map the occurrence of potential adverse effects and allow the assessment of patient satisfaction. Finally, it is worth noting the relevance of professional supervision for bleaching treatments due to the toxicity of hydrogen peroxide, which may cause gingival irritation if the agent directly contacts the gingiva (31) or inflammatory reactions in the digestive tract if the product is accidentally ingested (32).

## Conclusions

Except for dentifrices, the color change outcomes of the evaluated over-the-counter bleaching treatments were as satisfactory as in-office bleaching, without differences between groups for gloss change. The present *in vitro* study suggests that the evaluated over-the-counter products did not influence the shear bond strength of orthodontic brackets to teeth.

## Figures and Tables

**Table 1:** Bleaching protocol used for each group.

Group	Bleaching Protocol
HP35	35% hydrogen peroxide gel (Whiteness HP – FGM, Joinville, SC, Brazil) was applied on the buccal surface (Positive control group). Three 45-minute sessions (three 15-minute applications according to the product manufacturer's recommendations) occurred with a 48-hour interval between sessions.
DHP	The samples were subjected to mechanical brushing cycles in a toothbrushing machine (Odeme Dental Research, Luzerna, SC, Brazil) programmed to perform 60 reciprocating brushing movements per minute with a 200g load. A suspension containing a 2% hydrogen peroxide dentifrice (Colgate Luminous White Advanced - Colgate - São Paulo, SP, Brazil) and distilled water, in a 1:2 ratio, in weigh was used during 850 cycles, simulating approximately 180 toothbrushing days using soft bristle toothbrushes (Colgate Classic – Colgate, São Paulo, SP, Brazil).
MouHP	The samples were immersed in a recipient with mouthwash containing 2.5% hydrogen peroxide (Listerine Whitening Extreme – Johnson and Johnson, São José dos Campos, SP, Brazil) for 12 hours, simulating 12 months of use.
PON	A paint-on gel containing 6% hydrogen peroxide (Whitening Pen – Dazzling White, Guangzhou, China) was applied during 10-minute for applications daily for 14 days.
DWHP	The samples were subjected to mechanical brushing cycles in a toothbrushing machine (Odeme Dental Research, Luzerna, SC, Brazil) programmed to perform 60 reciprocating brushing movements per minute with a 200g load. A A suspension containing an conventional dentifrice without hydrogen peroxide (Negative control - Colgate Total 12 - Colgate - São Paulo, SP, Brazil) and distilled water, in a 1:2 ratio, in weigh was used during 850 cycles, simulating approximately 180 toothbrushing days using soft bristle toothbrushes (Colgate Classic – Colgate, São Paulo, SP, Brazil).

**Table 2:** Comparison between groups for ΔE00 and ΔEab values.

Groups	ΔE_00_ (median, minimum-maximum value)	ΔE_ab_ (median, minimum-maximum value)
HP35	11.09 (6.17 – 12.60)^A^	16.38 (8.87 – 18.50)^A^
MouHP	10.47 (8.01 – 13.81)^A^	15.26 (1.41 – 19.46)^A^
DWHP	8.49 (4.15 – 12.47)^B^	11.45 (6.04 – 17.45)^B^
DHP	7.75 (2.06 – 10.83)^B^	11.17 (2.67 – 16.12)^B^
PON	10.24 (1.99 – 12.92)^AB^	14.40 (2.20 – 18.60)^AB^

**Table 3:** Comparison between groups for gloss values.

Groups	Gloss change (mean±standard deviation)
HP35	6.39 ± 1.20^A^
MouHP	5.17 ± 2.06^A^
DWHP	5.76 ± 1.14^A^
DHP	5.52 ± 1.32^A^
PON	5.64 ± 1.84^A^

**Table 4:** Comparison between groups for shear values.

Groups	Shear (median, minimum-maximum value)
HP35	202.0 (65– 357)A
MouHP	185.0 (96 - 364)A
DWHP	188.0 (66 – 421)A
DHP	126.0 (79 – 449)A
PON	168.5 (74 – 335)A

## Data Availability

The datasets used and/or analyzed during the current study are available from the corresponding author.
